# A systematic classification of megakaryocytic dysplasia and its impact on prognosis for patients with myelodysplastic syndromes

**DOI:** 10.1186/s40164-016-0041-6

**Published:** 2016-04-27

**Authors:** Gege Feng, Robert Peter Gale, Wen Cui, Wenyu Cai, Gang Huang, Zefeng Xu, Tiejun Qin, Yue Zhang, Bing Li, Liwei Fang, Hongli Zhang, Lijuan Pan, Naibo Hu, Shiqiang Qu, Jingya Wang, Yajuan Cui, Zhijian Xiao

**Affiliations:** 1 MDS and MPN Centre, Institute of Hematology and Blood Diseases Hospital, Chinese Academy of Medical Sciences & Peking Union Medical College, 288 Nanjing Road, Tianjin, 300020 China; 2 State Key Laboratory of Experimental Hematology, Institute of Hematology and Blood Diseases Hospital, Chinese Academy of Medical Sciences & Peking Union Medical College, Tianjin, China; 3 Hematology Research Center, Division of Experimental Medicine, Department of Medicine, Imperial College London, London, UK; 4 Department of Pathology, Institute of Hematology and Blood Diseases Hospital, Chinese Academy of Medical Sciences & Peking Union Medical College, Tianjin, China; 5 Divisions of Experimental Hematology and Cancer Biology, Cincinnati Children’s Hospital Medical Center, Cincinnati, OH USA

**Keywords:** Dysplastic megakaryocytes, MDS, Immunochemistry, Prognosis

## Abstract

**Background:**

Dys-megakaryopoiesis is defined as ≥10 % of dysplastic megakaryocytes in bone marrow smears by the World Health Organization. However, concordance rates for dysplastic megakaryocytes between different observers is low and, consequently, evaluation of dysmegakaryopoiesis is also often discordant.

**Results:**

We performed CD41 immune staining and proposed a systematic classification of dys-megakaryopoiesis on bone marrow films: (1) micro-megakaryocytes (<12 µm); (2) micro-megakaryocytes (12–40 µm) with 1 nucleus; (3) micro-megakaryocytes (12–40 µm) with 2 nuclei; (4) micro-megakaryocytes (12–40 um) with multiple (more than 2) nuclei; (5) dysplastic megakaryocytes (≥40 µm) with 1 nucleus; (6) dysplastic megakaryocytes (≥40 µm) with 2 nuclei; and (7) dysplastic megakaryocytes (≥40 µm) with multiple (more than 2) nuclei. Further, we evaluated the prognostic impact of micro-megakaryocytes and dysplastic mono-nucleated megakaryocytes on MDS patients. The best discriminator cut-off point for each group was determined by the minimal *P* value approach. In multivariate analyses micro-megakaryocytes ≥25 % and dysplastic mono-nucleated megakaryocytes ≥30 % were independent adverse prognostic factors (hazard ratio [HR] = 1.58 [95 % confidence interval [CI], 1.11, 2.23]; P = 0.010 and 1.53 [1.09, 2.16]; P = 0.014).

**Conclusions:**

Our data suggest integration of micro-megakaryocytes and dysplastic mono-nucleated megakaryocytes improve predictive accuracy of the international prognostic scoring system-revised (IPSS-R) scoring system.

**Electronic supplementary material:**

The online version of this article (doi:10.1186/s40164-016-0041-6) contains supplementary material, which is available to authorized users.

## Background

Myelodysplastic syndromes (MDS) are a heterogeneous group of bone marrow neoplasms with variable clinical courses and prognoses [1]. Distinguishing the different form of MDS is important for accurate diagnosis, predicting outcomes and directing therapy. Several variables are used to distinguish different forms of MDS including morphology, histology, blood and bone marrow cell counts, cytogenetics and molecular genetics. Despite recent advances, cytological features in blood films and bone marrow aspirates and histological findings in trephine biopsies remain key elements for diagnosing MDS [2, 3]. Among the histological parameters of MDS, multi-lineage dysplasia and percent bone marrow blasts are associated with unfavorable outcomes [4–7].

Megakaryocyte morphology is another important component in classifying MDS. The World Health Organization (WHO) 2008 classification defines dys-megakaryopoiesis as micro-megakaryocytes, hypo-lobed, or non-lobed nuclei in megakaryocytes of all sizes and multiple, widely-separated nuclei [8]. Although this definition of dys-megakaryopoiesis is potentially useful, there is no precise definition of micro-megakaryocytes in the WHO classification. Consequently it is not surprising that there is low concordance amongst observers for micro-megakaryocytes in bone marrow samples from persons with MDS [4, 9–12].

Megakaryocytes express surface CD41/CD61 and/or CD42b and CD42a [13, 14]. The glycoprotein (Gp) IIb (CD41), which has been considered a specific marker for the megakaryocyte lineage [15], can be detected during megakaryocytic differentiation at a stage of a late megakaryocytic progenitor [16–18]. Consequently, using CD41 to identify megakaryocytes may be a better way to define dysplastic megakaryocytes than Wright-Giemsa or May-Grünwald-Giemsa staining. We used CD41 immune staining to identify megakaryocytes and assess if they were dysplastic in bone marrow smears from persons with MDS. Further, we tried to describe the morphological features of megakaryocytic dysplasia by developing a systematic classification of megakaryocytic dysplasia and analyze the impact of our classification of dys-megakaryopoiesis on determining the prognosis of persons with MDS.

## Methods

### Study cohort

The study was approved by the ethics committees of the institute of hematology, Chinese Academy Of Medical Sciences (CAMS) and Peking Union Medical College (PUMC) according to guidelines of the declaration of Helsinki. In this retrospective analysis, the study cohort included 422 consecutive new-diagnosed subjects that were seen at the Institute of Hematology and Blood Disease Hospital, Chinese Academy of Medical Sciences from January, 2000 to April, 2014. 8 subsequently received a haematopoietic cell transplant, 14, decitabine, 45, other chemotherapy and the remainder cyclosporine or thalidomide and best supportive care. Cases were re-reviewed by two blinded pathologists (W Cui and W Cai) and classified using the 2008 WHO criteria [2]. Subjects with suspected therapy-related MDS were excluded as the clinical course was typically progressive and treatment with conventional therapy was usually associated with a poor prognosis [19]. Furthermore, there was no Down Syndrome patient in the cohort. Follow-up data were available for 370 subjects (88 %). Date of last follow-up was December 15, 2014 or date of last contact. Median follow-up was 22 months (range 1–180 months). Subjects with lower-risk MDS fall into the international prognostic scoring system-revised (IPSS-R) categories of very low-, low-, and intermediate-risk groups and those with higher-risk MDS into the high- and very high-risk groups [20].

### Cytologic analysis

Bone marrow smears from diagnosis were reviewed using an avidin–biotin-complex method (ABC; CD41 immune staining) by the experts who were blinded for patients’ diagnoses, cytopenias and cytogenetic status in cytology. The preparation of bone marrow smear was a relatively uniform procedure. The marrow area on every smear was approximate to 1.5 × 3.0 cm with proper and relatively uniform thickness. ≥30 megakaryocytes were evaluated and the frequency of morphologic abnormalities was recorded. The presence of nuclear hypolobation, single or multiple separate small round nuclei were considered as main characteristics of dys-megakaryocytopoiesis.

### Statistical analyses

Statistical analyses were performed using SPSS 19.0 software or SAS software. The best discriminator threshold was detected using the minimal P value approach (a method aimed at minimizing the identification of rare classes of subjects) and considering survival (log-rank statistic) as the dependent variable [21, 22]. The functional form of the covariate under study was also evaluated using Martingale residual analysis [23].

Numerical variables were summarized by median and range. Categorical variables were described with count and relative frequency (%) of subjects in each category. Comparison of numerical variables between groups was carried out using a non-parametric approach (Mann–Whitney test). Comparison of the distribution of categorical variables in different groups was performed with χ2 test (unordered categorical variable) or the non-parametric approach (ordinal categorical variable).

Median survival was estimated using the Kaplan–Meier method and compared using the log-rank test. Cox proportional hazard regression model was used for multivariate analyses. P values were two-tailed and statistical significance was set as the level of P < 0.05.

## Results

### Subjects variables

Median age at diagnosis was 50 years (range 16–83 years). 286 subjects (68 %) were male. Distribution of WHO subtypes, IPSS-R cytogenetic category and IPSS-R classification are indicated in Table 1.

**Table 1:** Clinical variables (N = 422)

	Subjects (n = 422)
Median age (range, years)	50 (16–83)
Male	286 (68 %)
WHO classification	
RA	22 (5 %)
RN	2
RT	3
RARS	24 (6 %)
RCMD	198 (47 %)
RAEB-1	84 (20 %)
RAEB-2	76 (18 %)
MDS-U	8
MDS with del(5q) only	5
IPSS-R cytogenetic category	
Very-good	6
Good	208 (49 %)
Intermediate	143 (34 %)
Poor	23 (5 %)
Very-poor	42 (10 %)
IPSS-R (%)	
Very-low	6
Low	99 (23 %)
Intermediate	135 (32 %)
High	105 (25 %)
Very-high	77 (18 %)

### Cyto-morphologic evaluation of megakaryocyte dysplasia

**Fig. 1:**
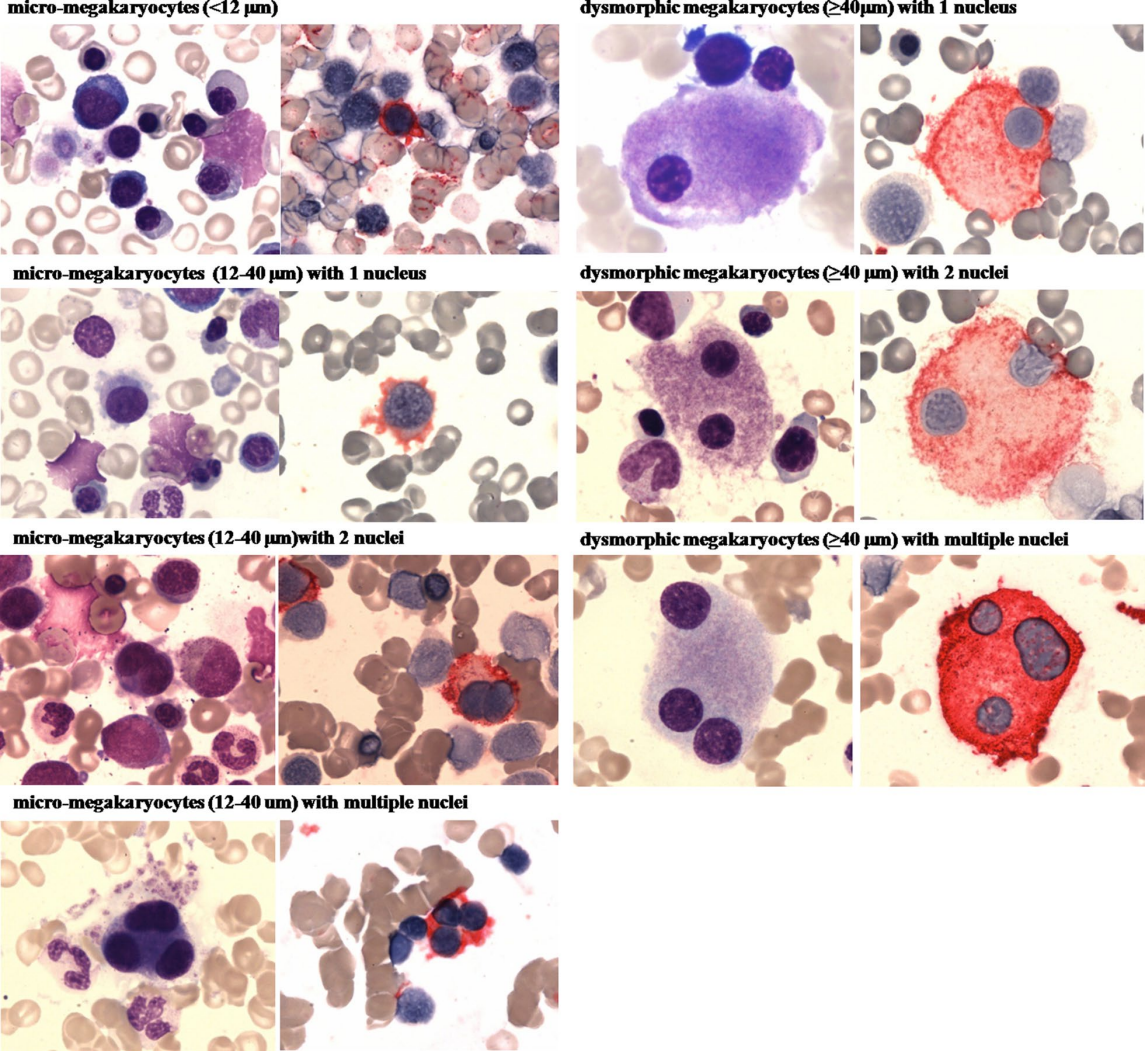
Wright-Giemsa staining and CD41 immune staining of dysplastic megakaryocytes on bone marrow smears

Megakaryocyte dysplasia was detected in 374 subjects (89 %). Median frequency of dysplastic megakaryocytes was 14 % (range 0–100 %). Patients without megakaryocytic dysplasia included RA (17 %), RARS (15 %), RCMD (42 %), RAEB-1 (17 %), RAEB-2 (6 %), MDS-U (4 %). Dysplastic megakaryocytes were assigned to 7 categories: (1) micro-megakaryocytes (<12 µm); (2) micro-megakaryocytes (12–40 µm) with 1 nucleus; (3) micro-megakaryocytes (12–40 µm) with 2 nuclei; (4) micro-megakaryocytes (12–40 um) with multiple (more than 2) nuclei; (5) dysplastic megakaryocytes (≥40 µm) with 1 nucleus; (6) dysplastic megakaryocytes (≥40 µm) with 2 nuclei; and (7) dysplastic megakaryocytes (≥40 µm) with multiple (more than 2) nuclei (Fig. 1). The most frequent dysplastic megakaryocytes were micro-megakaryocytes (12–40 µm) with 1 nucleus and dysplastic megakaryocytes (≥40 µm) with 1 nucleus, with median frequency of 28 % (0–91 %) and 29 % (0–78 %), respectively. Distribution of each type is shown in Additional file 1: Figure S1.

To analyze the prognostic impact of dys-megakaryopoiesis, we identified the cutoff point for the two prognostic classes with the greatest differences according to the smallest P value for micro-megakaryocytes at 25 % as well as mono-nucleated dys-megakaryopoiesis at 30 %. Subjects without megakaryocytic dysplasia were all grouped to micro-megakaryocytes <25 % and mono-nucleated dys-megakaryopoiesis <30 %.

### Association between subject variables and megakaryocyte dysplasia

Clinical and laboratory variables in subjects with micro-megakaryocytes <25 and ≥25 % are compared in Additional file 1: Table S1. A similar comparison between subjects with mono-nucleated dys-megakaryopoiesis <30 and ≥30 % is outlined in Additional file 1: Table S2.

Increased micro-megakaryocytes and mono-nucleated dys-megakaryopoiesis were significantly associated with lower levels of platelet count (P < 0.001 and P < 0.001) and higher levels of bone marrow blasts (P < 0.001 and P < 0.001). Distributions of WHO 2008 subtypes (P = 0.001 and P < 0.001), IPSS-R cytogenetic category (P = 0.002 and P = 0.001) and IPSS-R risk cohorts (P < 0.001 and P < 0.001) were also significantly different. There was no significant difference in age, gender, hemoglobin concentration and blood neutrophil counts at diagnosis between the two groups. In addition, levels of micro-megakaryocytes and dysplastic mono-nucleated megakaryocytes were significantly associated with abnormal karyotype (P = 0.026 and P = 0.014), complex karyotype (CK) (P = 0.034 and P = 0.022) and chromosome 7 aberrations (P = 0.004 and P = 0.003), but not with monosomal karyotype (MK) or del(5q) (Additional file 1: Table S3).

### Prognostic implications of megakaryocyte dysplasia

**Fig. 2:**
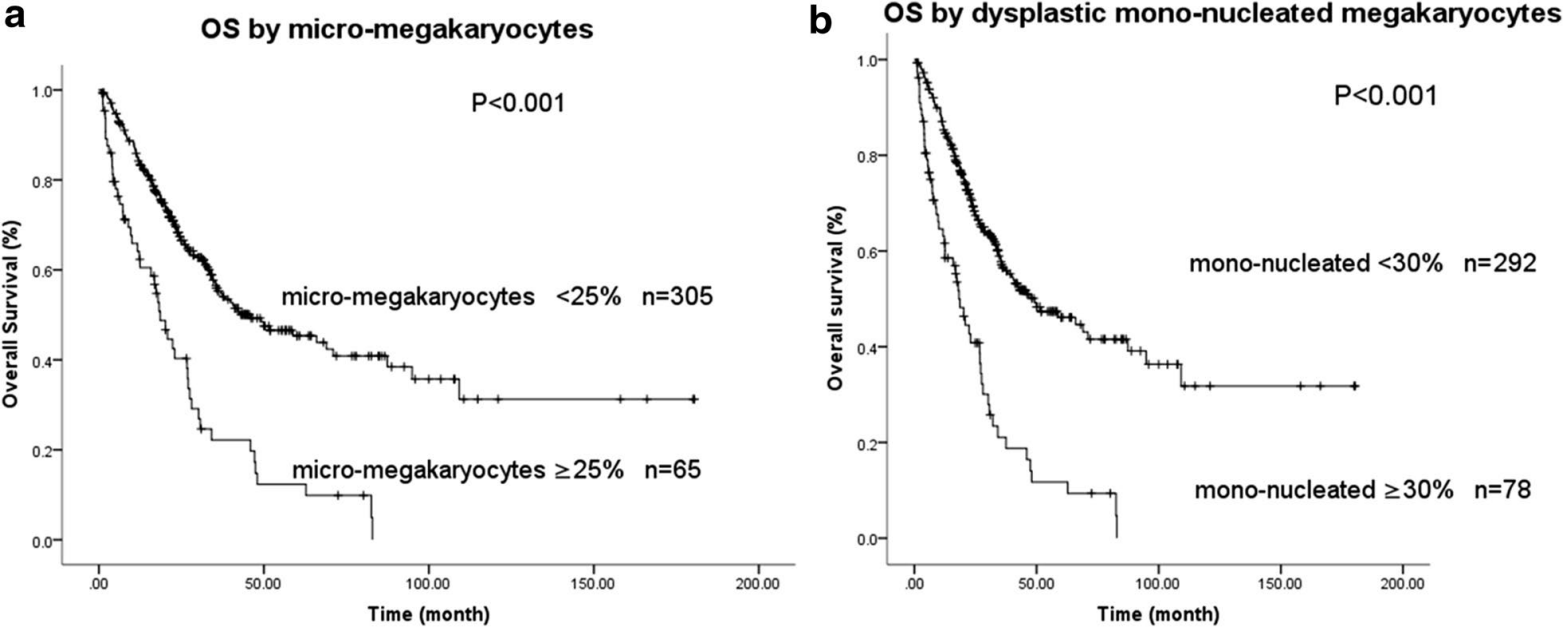
**a** Survival of 370 subjects with micro-megakaryocyte <25 or ≥25 %; **b** Survival of subjects with dysplastic mono-nucleated megakaryocytes <30 or ≥30 %

In univariate analyses, subjects with micro-megakaryocytes ≥25 % had poorer survival (median, 19 months [95 % CI 14–23 months]) than those with micro-megakaryocytes <25 % (46 months [28–64 months], P < 0.001; Fig. 2a). Similarly, patients with dysplastic mono-nucleated megakaryocytes ≥30 % demonstrated poorer survival as compared to those with dysplastic mono-nucleated megakaryocytes <30 % (18 months [14–23 months] vs. 49 months [31–68 months], P < 0.001; Fig. 2b). Other significant predictors of survival in univariate analyses were male gender (P = 0.009), age ≥60 years (P < 0.001), hemoglobin concentration <80 g/L (P = 0.001), neutrophils <0.8 × 10E + 9/L (P = 0.001), platelets <50 × 10E + 9/L (P = 0.002), bone marrow blasts >10 % (P < 0.001), IPSS-R cytogenetic category (P < 0.001) and IPSS-R score (P < 0.001).

We performed a multivariate Cox regression model including gender, age, micro-megakaryocytes and IPSS-R. Male gender (hazard ratio [HR] = 1.5; 95 % CI 1.0–2.0; P = 0.029), age ≥60 years (HR = 1.5; [1.1–2.0]; P = 0.016), micro-megakaryocytes ≥25 % (HR = 1.6 [1.1–2.2]; P = 0.010) and IPSS-R score (P < 0.001) were significantly associated with survival (Table 2). In a similar analysis including gender, age, dysplastic mono-nucleated megakaryocytes and IPSS-R, only male gender (HR = 1.5; 95 % CI 1.0–2.0; P = 0.028), age ≥60 years (HR = 1.5; 95 % CI 1.1–2.0; P = 0.018), dysplastic mono-nucleated megakaryocytes ≥30 % (HR = 1.533; [1.1–2.2]; P = 0.014) and IPSS-R (P < 0.001) remained in the final model (Table 3).

**Table 2:** Multivariate Cox regression analysis with respect to micro-megakaryocytes

	HR	95 % CI	P value
Gender			0.029
Male	1.458	1.039–2.047	
Female*	1.0		
Age (year)			0.016
≥60	1.485	1.078–2.047	
<60*	1.0		
IPSS-R			<0.001
Very-low	0	0–2.31E133	
Low	0.106	0.062–0.180	
Intermediate	0.227	0.151–0.341	
High	0.597	0.410–0.869	
Very-high*	1.0		
Micro-megakaryocytes (%)			0.010
≥25	1.575	1.113–2.230	
<25*	1.0	1.0	

* The reference

**Table 3:** Multivariate Cox regression analysis with respect to dysplastic mono-nucleated megakaryocytes

	HR	95 % CI	P value
Gender			0.028
Male	1.465	1.042–2.058	
Female*	1.0		
Age (year)			0.018
≥60	1.474	1.068–2.034	
<60*	1.0	–	
IPSS-R			<0.001
Very-low	0	0–5.24E132	
Low	0.108	0.063–0.185	
Intermediate	0.235	0.155–0.355	
High	0.607	0.416–0.885	
Very-high*	1.0		
Mono-nucleated (%)			0.014
≥30	1.533	1.089–2.160	
<30*	1.0		

* The reference

### Prognostic implications of megakaryocytic dysplasia in IPSS-R lower-risk subjects according to the IPSS-R score

**Fig. 3:**
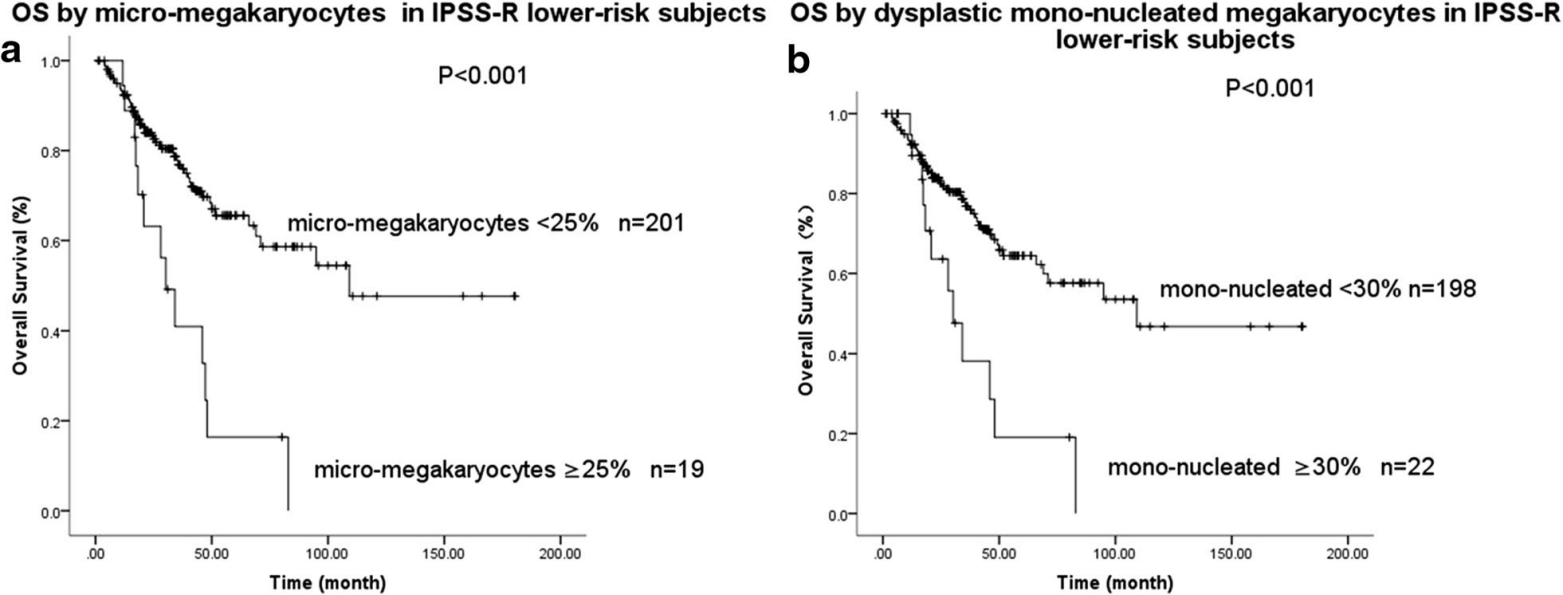
**a** Survival of subjects with IPSS-R lower-risk with micro-megakaryocyte <25 or ≥25 %; **b** survival of subjects with dysplastic mono-nucleated megakaryocytes <30 or ≥30 %

In the IPSS-R lower-risk subjects, those with micro-megakaryocytes ≥25 % had a poorer survival than patients with micro-megakaryocytes <25 % (P < 0.001; Fig. 3a). Similarly, there was a significant difference in survival between patients with mono-nucleated dys-megakaryopoiesis <30 and ≥30 % (P < 0.001; Fig. 3b). This association was not significant in subjects in the IPSS-R higher-risk cohort. Consequently, we performed stratified multivariate analyses to further evaluate prognostic implications of megakaryocytic dysplasia in IPSS-R lower-risk subjects only. In the model of cell size including age, gender, IPSS-R and micro-megakaryocytes, only age, IPSS-R and micro-megakaryocytes were significantly correlated with survival (Table 4). In a similar analysis considering dysplastic mono-nucleated megakaryocytes, only age, IPSS-R and dysplastic mono-nucleated megakaryocytes were significantly correlated with survival (Table 5).

**Table 4:** Multivariate analysis of the survival of IPSS-R lower-risk subjects with respect to micro-megakaryocytes

	HR	95 % CI	P value
Age (year)			0.024
≥60	1.856	1.086–3.171	
<60*	1.0		
Gender			0.185
IPSS-R			0.026
Very-low	0	0–6.17E272	
Low	0.478	0.280–0.816	
Intermediate*	1.0		
Micro-megakaryocytes (%)			<0.01
≥25	2.806	1.509–5.218	
<25*	1.0		

* The reference

**Table 5:** Multivariate analysis of the survival of IPSS-R lower-risk subjects with respect to dysplastic mono-nucleated megakaryocytes

	HR	95 % CI	P value
Age (year)			0.03
≥60	1.808	1.058–3.089	
<60*	1.0		
Gender			0.161
IPSS-R			0.018
Very-low	0	0–1.07E266	
Low	0.463	0.272–0.789	
Intermediate*	1.0		
Mono-nucleated (%)			<0.01
≥30	2.672	1.410–5.064	
<30*	1.0		

* The reference

## Discussion

In the WHO classification of myeloid neoplasms bone marrow dysplasia ≥10 % of the cells of a specific myeloid lineage is the cardinal diagnostic feature of the MDS. However, morphology is a subjective parameter under the routine staining conditions. For example, concordance for dys-megakaryopoiesis amongst observers was less than concordance for dys-granulopoiesis and dys-erythropoiesis [4]. Thus, specific immune staining may be a better way to evaluate megakaryocytes than routine histological methods [24].

Based on data from our study we propose a systematic classification of dys megakaryopoiesis on bone marrow films of persons with suspected MDS using CD41 immune staining. A study has reviewed marrow smears of 26 RA and 28 RAEB patients, and micro-megakaryocytes were identified in 39.3 and 46.5 % cases under Wright-Giemsa staining, compared with 92.3 and 100 % under CD41 immune staining (χ2 test: P < 0.005 and P = 0.01) (Wenyu Cai, unpublished data).

In our study, a larger number of dysplastic megakaryocytes was significantly associated with decreased platelet count, increased bone marrow blasts and increased IPSS-R scores. Cytogenetic abnormalities are associated with characteristic dysplastic features, e.g. isolated del(5q) and hypo-lobed and un-lobed megakaryocyte nuclei and del(17p) with hypo-lobed neutrophil nuclei [25]. Data from our study also indicated parallel increases in micro-megakaryocytes and dysplastic mono-nucleated megakaryocytes and IPSS-R cytogenetic scores, as well as frequencies of abnormal karyotype, CK and chromosome 7 aberrations. However, according to our study, there was no significant association of dysplastic megakaryocytes with del(5q). Hypolobulation is also commonly seen in all other subtypes besides MDS with del(5q) only. This unexpected result may be on account of the small proportion of del(5q) patients in our study cohort (5/422). A larger dataset is necessary for further research on this issue. Furthermore, we suspect that abnormalities in process of endo-reduplication may play a more important role than aberrant endomitosis in dysplastic megakaryocytes, for micro-megakaryocytes with 1 nucleus and dysplastic megakaryocytes (≥40 µm) with 1 nucleus were most frequently seen in our study.

Studies reported a threshold of 10 % dysplastic granulocytes as well as 30–40 % dysplastic megakaryocytes as the best survival discriminator [4, 11, 26, 27]. We found a threshold of 25 % of micro-megakaryocytes and 30 % of dysplastic mono-nucleated megakaryocytes as the best survival discriminators, which was independent of age, gender and IPSS-R risk score. We also found micro-megakaryocytes and dysplastic mono-nucleated megakaryocytes were independently associated with survival in subjects with IPSS-R lower-risk MDS. Based on these data we suggest that adding these variables to the IPSS-R model could improve the predictive accuracy in untreated subjects and those with IPSS-R lower-risk MDS. External validation of concordance between observers using our technique and dysplastic megakaryocytes classification is needed.

## Additional file

**Additional file 1.** Distribution of dysplastic megakaryocytes in subjects and variables of subjects with dysplastic megakaryocytes.
