# Supplementary material for: A systematic classification of megakaryocytic dysplasia and its impact on prognosis for patients with myelodysplastic syndromes
Source: Exp Hematol Oncol. 2016 Apr 27;5:12. doi: 10.1186/s40164-016-0041-6 (PMC4848808; doi:10.1186/s40164-016-0041-6)

Table SI. Variables of subjects with micro-megakaryocyte <25% and ≥25%

|  | micro-megakaryocyte <25% | micro-megakaryocyte ≥25% | P-value |
| --- | --- | --- | --- |
| N | 343 (81) | 79 (19) |  |
| Gender  Male | 234 (68%) | 52 (66%) | 0.681 |
| Age (year)  Median (range) | 50 (16-83) | 48 (18-76) | 0.471 |
| ANC×10E+9/L  Median (range) | 1.10 (0-13) | 0.97 (0-7) | 0.121 |
| Hemoglobin (g/L)  Median (range) | 74 (28-150) | 72 (25-157) | 0.682 |
| Platelets×10E+9/L  Median (range) | 95 (6-1396) | 35 (2-554) | <0.001 |
| Bone marrow blasts (%)  Median (range) | 3 (0-19.5) | 6 (0-19.5) | <0.001 |
| WHO 2008 classification  RA  RN  RT  RARS  RCMD  RAEB-1  RAEB-2  MDS-U  MDS with del(5q) only | 22 (6%)  2  3  24 (7%)  164 (48%)  66 (19%)  50 (15)  7  5 | 0  0  0  0  34 (43%)  18 (23%)  26 (33%)  1  0 | 0.001 |
| IPSS-R cytogenetic category  Very-good  Good  Intermediate  Poor  Very-poor | 6  178 (52%)  115 (34%)  14 (4%)  30 (9%) | 0  30 (38)  28 (35)  9 (11)  12 (15) | 0.002 |
| IPSS-R  Very-low  Low  Intermediate  High  Very-high | 6  90(26%)  121 (35%)  79 (23%)  47 (14%) | 0  9 (11%)  14 (18%)  26 (33%)  30 (38%) | <0.001 |

Table SII. Variables of subjects with mono-nucleated dys-megakaryopoiesis <30% and ≥30%

|  | mono-nucleated dys-megakaryopoiesis <30% | mono-nucleated dys-megakaryopoiesis ≥30% | P-value |
| --- | --- | --- | --- |
| N | 327 (77) | 95 (23) |  |
| Male | 223 (68) | 63 (66) | 0.730 |
| Age (year)  Median (range) | 49 (16-83) | 51 (18-76) | 0.784 |
| ANC, ×10E+9/L  Median (range) | 1.12 (0-13) | 0.93 (0-7) | 0.074 |
| Hemoglobin (g/L)  Median (range) | 74 (28-152) | 72 (25-157) | 0.751 |
| Platelets, ×10E+9/L  Median (range) | 101 (6-1396) | 38 (2-554) | <0.001 |
| Bone marrow blasts (%)  Median (range) | 3 (0-19.5) | 6 (0-19) | <0.001 |
| WHO 2008 classification  RA  RN  RT  RARS  RCMD  RAEB-1  RAEB-2  MDS-U  MDS with del(5q) only | 22 (7%)  2  3  23 (7%)  162 (50%)  62 (19%)  41 (13%)  7  5 | 0  0  0  1  36 (38%)  22 (23%)  35 (37%)  1  0 | <0.001 |
| IPSS-R cytogenetic category  Very-good  Good  Intermediate  Poor  Very-poor | 6  171 (52%)  109 (33%)  14 (4%)  27 (8%) | 0  37 (39%)  34 (36%)  9 (10%)  15 (16%) | 0.001 |
| IPSS-R  Very-low  Low  Intermediate  High  Very-high | 6  89 (27%)  119 (36%)  74 (23%)  39 (12%) | 0  10 (11%)  16 (17%)  31 (33%)  38 (40%) | <0.001 |

Table SIII. Cytogenetic abnormalities of subjects in low- and high-level cohorts of micro-megakaryocytes and mono-nucleated dys-megakaryopoiesis

|  | All subjects, n(%) | Micro-megakaryocytes, n (%) | | | Mono-nucleated dys-megakaryopoiesis, n (%) | | |
| --- | --- | --- | --- | --- | --- | --- | --- |
|  |  | **<25%** | **≥25%** | **P** | **<30%** | **≥30%** | **P** |
| Karyotype  Normal  Abnormal  MK  CK | 175 (41)  247 (59)  43 (10)  55 (13) | 151 (44)  192 (56)  31 (9)  39 (11) | 24 (30)  55 (70)  12 (15)  16 (20) | 0.026  0.103  0.034 | 146(45)  181(55)  29 (9)  36(11) | 29 (31)  66 (69)  14 (15)  19 (20) | 0.014  0.096  0.022 |
| chromosome 7  Normal  Abnormal | 387 (92)  35 (8) | 321 (94)  22 (6) | 66 (83)  13 (17) | 0.004 | 307 (94)  20 (6) | 80 (84)  15 (16) | 0.003 |
| Del (5q)  yes  no | 26 (6)  396 (94) | 21 (6)  322 (94) | 5 (6)  74 (94) | 0.945 | 19 (6)  308 (94) | 7 (7)  88 (93) | 0.578 |

complex karyotype; MK, monosomal karyotype;

Figure legends

Figure SI. Distribution of dys-megakaryopoiesis in all subjects and subjects of each WHO cohort.


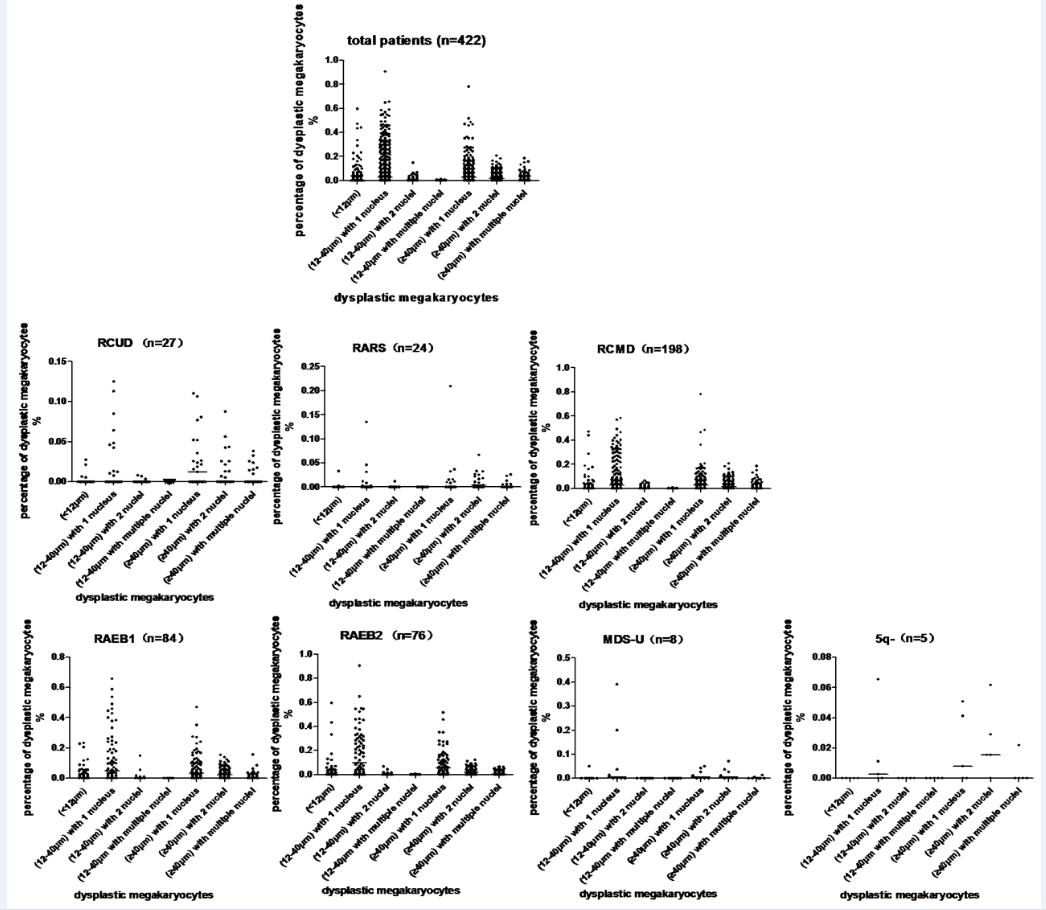

Supplement: Supplementary file 1 — Additional file 1. Distribution of dysplastic megakaryocytes in subjects and variables of subjects with dysplastic megakaryocytes. [file 40164_2016_41_MOESM1_ESM.docx]
